# The clinical and genetic profiles of spinal muscular atrophy with respiratory distress type 1 and identification of a novel mutation in *IGHMBP2* in China

**DOI:** 10.3389/fgene.2025.1594265

**Published:** 2025-07-04

**Authors:** Yanjun Wang, Ya Yang, Jingjing Wang, Qian Han, Nana Zhai, Shufang Xiao

**Affiliations:** Pediatric Intensive Care Unit, Kunming Children’s Hospital, Children’s Hospital Affiliated to Kunming Medical University, Kunming, Yunnan, China

**Keywords:** SMARD1, whole-exome sequencing, IGHMBP2, mutation spectrum, clinical profiles

## Abstract

**Background:**

Spinal muscular atrophy with respiratory distress type 1 (SMARD1, OMIM #604320) is a rare autosomal recessive hereditary degenerative motor neuron disease caused by mutations in IGHMBP2. There is a lack of data from China. This study investigated the clinical characteristics and genetic roots of SMARD1 patients.

**Methods:**

Routine detailed clinical assessments, laboratory examinations, and imaging assays were performed. Genetic variations in the families were investigated using whole-exome sequencing and Sanger sequencing, and then bioinformatic analyses were performed on the identified variant.

**Results:**

Here, we describe three female patients with SMARD1 from three unrelated families carrying compound heterozygous mutations in the *IGHMBP2* gene, which were inherited from both parents. Six mutations including a novel one (c.716T>C/p.L239P) were identified. Multiple lines of bioinformatic evidence suggested that the novel mutation was a likely detrimental variant. The c.1060G>A/p.G354S mutation was detected in both P1 and P3 and may be a hotspot in the Chinese population. Clinical presentations included delay in development, respiratory failure, hypotonia, distal limb muscle weakness, and diaphragm eventration or paralysis. Additionally, the variants identified in this study were compiled from relevant literature to analyze disease etiology, finding a distinctive distribution of genotypes across the severity of disease manifestations.

**Conclusion:**

This study broadened the knowledge on the genetic profile of SMARD1, improved pediatricians’ awareness of early identification and diagnosis, and offers useful data for patient clinical management.

## 1 Introduction

Spinal muscular atrophy with respiratory distress type 1 (SMARD1, OMIM #604320), also known as distal spinal muscular atrophy 1 (DSMA1) or distal hereditary motor neuropathy type Ⅵ (dHMN6), is a rare autosomal recessive hereditary degenerative motor neuron disease (Eckart et al., 2012). The pathogenic cause is the immunoglobulin μ-binding protein 2 gene (*IGHMBP2*, OMIM #600502), which is located on chromosome 11q13.2–q13.4. The *IGHMBP2* gene encodes for a 5′→3 ′ RNA helicase/ATPase and eventually causes loss of helicase function. This abnormality leads to dysfunction, degeneration, and loss of α-motor neurons in the ventral horn of the spinal cord, which ultimately causes atrophy of skeletal muscle fibers in the extremities, trunk, and diaphragm (Jankowsky et al., 2011; Grohmann et al., 2001; Jablonka and Yildirim, 2024). SMARD1 was first reported by Mellins et al. (1974) in 1974, and there is no exact prevalence. However, the prevalence is estimated to be less than 1/1,000,000 according to Orpha.net (https://www.orpha.net/en/disease/detail/98920). To date, approximately 150 patients have been reported (Viguier et al., 2019; Pekuz et al., 2022; Porro et al., 2014). According to the Human Gene Mutation Database (HGMD), a total of 110 variants associated with SMARD1 were reported. Among them, 78/110 (71%) are missense mutations, 19 are single or several bases deletion, 9 are intronic mutations, two are duplications, and two are large fragment deletions. Biallelic variants in *IGHMBP2* can also present with axonal Charcot–Marie–Tooth disease type 2S (CMT2S) (Cottenie et al., 2014).

The analysis of clinical and genetic profiles of the condition is particularly important due to the limited number of cases and multiple clinical phenotypes. The main clinical manifestations of SMARD1 are progressive distal limb muscle weakness, muscle atrophy, joint contracture, autonomic nerve dysfunction, and respiratory failure caused by diaphragmatic paralysis (Perego et al., 2020). The patients usually have a history of intrauterine growth retardation, low birth weight, weak crying, feeding difficulties, and delayed growth and development. They are characterized by inspiratory stridor, recurrent dyspnea or apnea, cyanosis, and the absence of deep tendon reflexes. Scoliosis, foot deformities, and joint contractures are common associated features (Perego et al., 2020). This disease needs to be differentiated from 5q spinal muscular atrophy type 1 (5q-SMA1) due to similar clinical features and a higher prevalence rate.

The overall median survival time of children who did not receive mechanical ventilation was only 5 months. The prognosis of children with respiratory support is difficult to estimate due to improvements in the quality of home ventilators (Viguier et al., 2019). Currently, the leading cause of death in patients is progressive neurological dysfunction of the autonomic nervous system and complications of respiratory therapy. There is no effective treatment for SMARD1, and research into gene therapy is ongoing (Perego et al., 2020; Saladini et al., 2020). The application of the first gene therapy approach for spinal muscular atrophy (SMA) linked with variants in survival motor neuron 1 (*SMN1*) provides potential strategies for intervention for SMARD1. In the future, gene therapy via gene replacement or gene correction may provide therapeutic targets to halt or possibly prevent neurodegenerative disease in SMARD1 patients.

Above all, there are significant challenges to early diagnosis, genetic counseling, and treatment in SMARD1. We report three rare cases of SMARD1 caused by *IGHMBP2* heterozygous mutations and confirm the association of a novel mutation with the disease, thus expanding the pathogenic gene profile and improving clinicians’ understanding of the condition.

## 2 Materials and methods

### 2.1 Patients

Thirty unrelated patients meeting SMA criteria underwent whole-exome sequencing screening to identify variants in the *IGHMBP2* gene. The age of the cases ranged from 1 month to 18 years. Consequently, three patients with SMARD1 were defined. This study was approved by the Ethics Committee of Kunming Children’s Hospital. Informed consent from the parents of the cases was obtained. After admission, the patients underwent detailed clinical assessments and laboratory examinations. Peripheral blood samples were collected from the patients and their parents for hematological and serum biochemistry examination. Imaging examinations included chest X-ray, color Doppler ultrasonography of the diaphragm, and brain MRI. Muscle strength was graded according to the Lovett muscle grading system.

### 2.2 Whole-exome sequencing (WES) and Sanger sequencing validation

We collected EDTA-anticoagulated whole blood samples from patients and their parents. The genomic DNA was extracted, and a genomic library was constructed using the Illumina standard protocol. A DNBSEQ-T7 sequencer (MGI, Shenzhen, China) was used to sequence the enriched library after the GenCap kit (MyGenostics, Beijing, China) captured the whole-exome target regions. Following quality control, BWA was used to map the clean reads to the UCSC hg19 human reference genome (Li and Durbin, 2009) (http://bio-bwa.sourceforge.net/). GATK (https://software.broadinstitute.org/gatk/) HaplotypeCaller identified the single-nucleotide polymorphism (SNP) and insertion/deletion (InDel) variations and then GATK VariantFiltration filtered for eligible variants.

All candidate mutations were confirmed by Sanger sequencing. Target genes were sequenced on an ABI prism 3,730 genetic analyzer (Applied Biosystems; Thermo Fisher Scientific). The variant sites were determined by comparing the DNA sequences with the corresponding GenBank (www.ncbi.nlm.nih.gov) reference sequences.

### 2.3 Bioinformatic prediction of variants

The pathogenicity of each variant was assessed in strict accordance with the American College of Medical Genetics and Genomics (ACMG) Standards and Guidelines (Richards et al., 2015) using Sorting Intolerant from Tolerant (SIFT, http://sift-dna.org), PolyPhen-2 (http://genetics.bwh.harvard.edu/pph2/index.shtml), and Mutation Taster (https://www.mutationtaster.org/). SpliceAI (https://spliceailookup.broadinstitute.org/) was performed to evaluate the pre-mRNA splicing impact.

### 2.4 3D modeling of the novel variant

SWISS-MODEL (Biasini et al., 2014) (https://swissmodel.expasy.org/interactive) was used for protein analysis. The visualization structure of the protein that caused the novel mutation was analyzed.

## 3 Results

### 3.1 Clinical presentations

Of the 30 unrelated patients meeting SMA criteria, the whole-exome sequencing screening identified compound heterozygous *IGHMBP2* variants in three patients with SMARD1. The major clinical features and genetic findings are summarized in Table 1.

**TABLE 1:** Clinical characteristics of the SMARD1 patients.

Variables	P1	P2	P3
	c.344C>T		
Genetic defect	c.1060G>A	c.1334A>C	c.716 T>C
	c.1061–2A>G	c.1666C>G	c.1060G>A
Sex	Female	Female	Female
Nationality	Han	Han	Yi
Age of onset of first symptoms	7 m	50 d	Birth
Age at the time of sample collection	1 y5 m	5 m	9 m
Fetal growth restriction	-	-	-
Premature birth	-	-	-
Feeble crying	-	+	+
Diaphragm eventration	+	+	-
Diaphragmatic paralysis	+	—	—
Respiratory system			
Onset of respiratory failure	1 y5 m	55 d	9 m
Tachypnea	+	+	+
Three depressions sign	+	+	+
Respiratory failure	+	+	+
Mechanical ventilation time	42 d	156 d	26 d
Hard to get off the ventilator	+	+	+
Respiratory pathogens	-	*Acinetobacter* *baumannii*, *pseudomonas aeruginosa*, *staphylococcus aureus*, *Candida* *glabrata*, and herpes	Parainfluenza virus and *Klebsiella* sp.
Tracheotomy	-	-	-
Arterial blood gas before mechanical ventilation			
PH	7.4	7.34	7.33
Oxygen partial pressure (mmHg)	45	60	58
Partial pressure of carbon dioxide (mmHg)	45	66	50
Neurological features			
Knee jerk	Absence	Absence	Absence
Hypomyotonia	+	+	+
Muscle strength grades	4, Lower extremities: grade 2	Lower extremities: grade 1–2	3, Lower extremities: grade 0
Neurogenic changes of EMG	+	—	—
Sensory conduction velocity slowing	+	—	—
Distal muscle atrophy	-	-	-
Scoliosis	-	-	-
Delay in development	+	+	+
Malnutrition	Severe	Severe	Mild
Arrhythmia	-	-	-
Strephenopodia	+	-	-
Intestinal dysfunction	+	+	-
Comorbidities	Myocardial damage	Heart failure, septic shock toxic encephalopathy, and pulmonary hypertension	-
Prognosis	Death at 1 y 6 m	Death at 7 m	Death at 10 m

Note: y, year; m, month; d, day; +, presence; -, absence.

#### 3.1.1 Patient 1

The female infant, the second child of a Han family, was born by Cesarean section at 38 weeks with a birth weight of 2.7 kg (-1SD) and no asphyxia at birth. Her mother conceived at the age of 42 years and was a high-risk maternal candidate. Her parents were non-consanguineous, healthy, and had no family history. The first child developed normally. At the age of 7 months, the infant was found to have developmental delay, such as occasionally turning over in the supine position, inability to bear weight in both lower limbs, inability to take objects flexibly, and inability to tear paper. The electromyography report showed extensive neurogenic lesions in the extremities, mainly axonal lesions of motor nerves, especially in the lower limbs. Brain MRI revealed no abnormalities. At the age of 1 year and 5 months, the child developed anhelation, dyspnea, cyanosis of the face and lips, food refusal, lethargy, and listlessness, and she was admitted to our department and was reported to have a body weight of 7.5 kg (-3SD).

**FIGURE 1:**
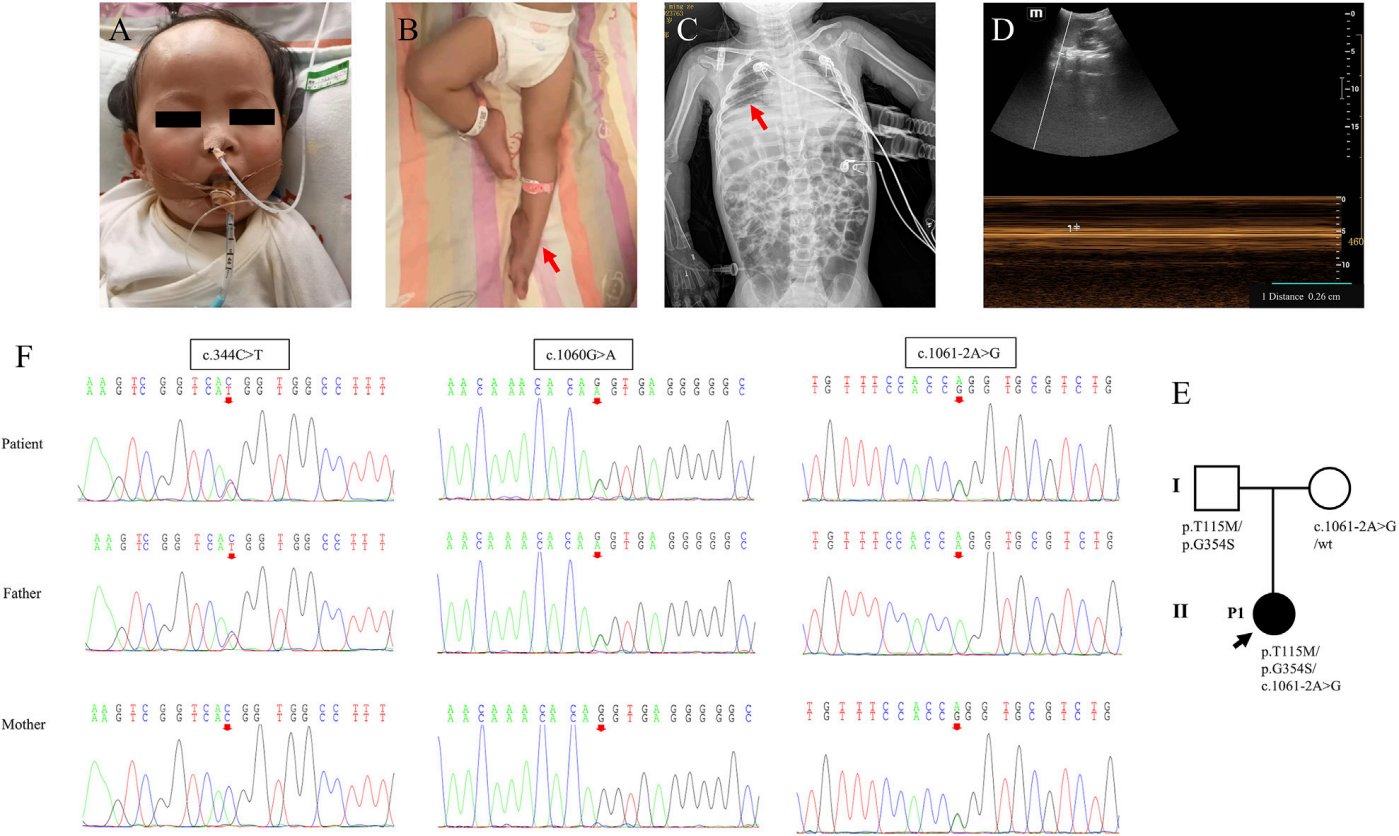
Clinical features and genetic results of P1. **(A,B)** Features of clinical symptoms. **(C)** The chest X-ray showed diaphragm eventration. **(D)** Diaphragmatic mobility ultrasound showed decreased diaphragmatic mobility distance of 0.26 cm (normal range: >0.5 cm). **(E)** Family pedigree. **(F)** Sanger sequencing results for the patient and her parents for the two mutations.

Physical examinations on admission showed that the patient presented with poor general condition, lethargic mental state, tachypnea (up to 65 breaths/minute), tachycardia (ranging from 160 to 170 beats/minute), and SPO2 85% under mask oxygen inhalation. She exhibited cyanosis of facial color and lips, nasal alar agitation, tachypnea, and suprasternal, intercostal, and subcostal retractions. Auscultation showed low breath sounds in both lungs and a little phlegm rale. The patient presented with hypotonia, where the muscle strength of upper limbs was grade 3–4 and muscle strength of the lower limbs was grade 2, foot varus, absent knee reflex, and negative pathological signs (Figures 1A,B; Table 1).

Laboratory examinations showed that troponin-T 47.23 pg/mL (normal range: 12.7–24.9 pg/mL) was elevated and that serum lactate, blood sugar, creatine kinase, creatine kinase-MB, hepatic function, renal function, myocardial enzyme, coagulation function, and serum electrolytes were in the normal range. Blood amino acids and urine organic acids were normal, but multiple acylcarnitine in blood was slightly increased, which returned to normal after 4 days, and threonine was slightly increased. Chest radiography revealed pneumonia and diaphragm eventration (Figure 1C). Diaphragmatic mobility ultrasound showed decreased diaphragmatic mobility. The electroencephalogram suggested increased δ and Ɵ wave activity. Electrocardiogram and abdominal color Doppler ultrasound were normal. Cardiac color Doppler ultrasound showed trace pericardial effusion.

The trios-WES and Sanger sequencing showed that the infant inherited compound heterozygous mutations in *IGHMBP2* from her parents, c.344C>T (p.T115M), c.1060G>A (p.G354S), and c.1061–2A>G. Her mother had the heterozygous mutation c.1061–2A>G, and her father had compound heterozygous mutations c.344C>T (p.T115M) and c.1060G>A (p.G354S) (Figure 1; Table 1). Her parents had no clinical symptoms.

After ventilator-assisted mechanical ventilation and combined anti-infection treatment, it was still difficult to wean the patient from mechanical ventilation. Then, the patient died at the age of 1 year and 6 months after the family decided to cease treatment.

#### 3.1.2 Patient 2

The female infant, the second child from a Han and non-consanguineous family, was born by Cesarean section at 40 weeks and 2 days with a birth weight of 2.9 kg and no asphyxia at birth. At the age of 50 days, the infant developed anorexia, listlessness, and lethargy, and then she developed progressive dyspnea, mouth breathing, and convulsions. The child was admitted to our department at the age of 2 months with a height of 57 cm (median), weight of 3.9 kg (-2SD), head circumference of 35 cm, and abdominal circumference of 33 cm.

**FIGURE 2:**
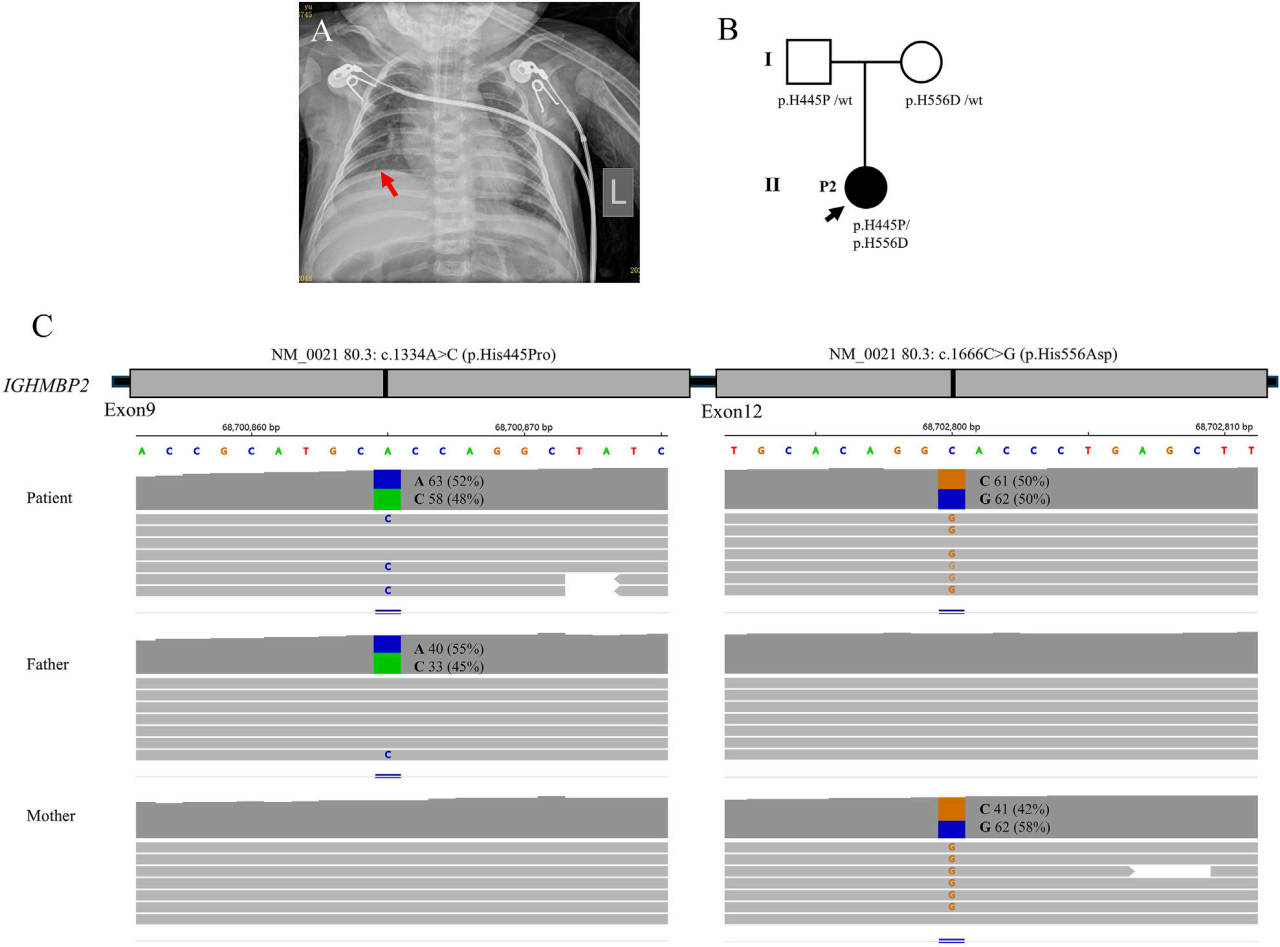
Clinical features and genetic results of P2. **(A)** The chest X-ray showed bilateral pneumonia and right diaphragm eventration. **(B)** Family pedigree. **(C)** Trios whole-exome sequencing results.

Physical examinations on admission showed that the infant exhibited poor general condition, lethargic mental state, tachypnea (up to 70 breaths/minute), tachycardia (ranging from 170 to 175 beats/minute), and SpO_2_ 80% under mask oxygen inhalation. She presented with cyanosis of facial color and lips, nasal alar agitation, tachypnea, and suprasternal, intercostal, and subcostal retractions. Auscultation of the lungs showed severe and fine wet rales that were detected in bilateral lungs. The infant presented with hypotonia, absent knee reflex, and negative pathological signs. The muscle strength grade was grade 3 for the upper extremities and grade 1–2 for the lower extremities (Figures 2A,B; Table 1).

Laboratory examinations showed that troponin-T 84.06 pg/mL (normal range: 12.7–24.9 pg/mL), lactic dehydrogenase 461 U/L (normal range: 67–394.1 U/L), aspartic transaminase 91 U/L (normal range: 0–40 U/L), and alanine aminotransaminase 112 U/L (normal range: 0–40 U/L) were elevated. Serum lactate, blood sugar, creatine kinase, creatine kinase-MB, cerebrospinal fluid examination, renal function, coagulation function, and urinary organic acids were all in the normal range. The chest radiography revealed bilateral pneumonia and right diaphragm eventration. Brain CT, electrocardiogram, electroencephalogram, cardiac color Doppler ultrasound, and abdominal color Doppler ultrasound showed no abnormalities. *Acinetobacter baumannii*, *Pseudomonas aeruginosa*, *Staphylococcus aureus*, *Candida glabrata*, and herpes virus were detected from the respiratory tract.

The trios-WES identified a heterozygous pathogenic variant c.1334A>C (p.H445P) and a heterozygous uncertain variant c.1666C>G (p.H556D) in *IGHMBP2*, finally establishing the genetic diagnosis of SMARD1. The WES study did not identify other suspected disease-causing variants. The carrier status was confirmed in her parents who had no clinical symptoms.

After admission, the patient was treated with mechanical ventilation for more than 5 months along with anti-infection therapy, and her lung condition improved, but she still could not be weaned from mechanical ventilation. Her family decided to cease treatment, and the infant died at the age of 7 months.

#### 3.1.3 Patient 3

The female infant, the second child of a Yi family and unrelated healthy parents, was delivered at term via Cesarean section at 37 weeks with a birth weight of 2.9 kg. There was no asphyxia at birth. The infant’s mother had a history of a scarred uterus and presented with hypertension after 30 weeks of pregnancy. The child was hospitalized at birth due to neonatal pneumonia and at the age of 5 months due to severe pneumonia. The patient was developmentally delayed and began to hold the head up at 5 months, began to sit at 8 months, still sat unsteadily at 9 months of age, and was unable to crawl, walk, or speak. The parents’ first child was a boy diagnosed with hepatoblastoma at the age of 3 years and 2 months and died at the age of 5 years after chemotherapy and partial hepatectomy. At the age of 9 months, the patient developed cough, respiratory stress, and cyanosis of the lips and was admitted to our department. The child’s height was 69 cm (-1SD), weight 7.5 kg (-1SD), head circumference 42 cm, chest circumference 45 cm, and abdominal circumference 37 cm.

**FIGURE 3:**
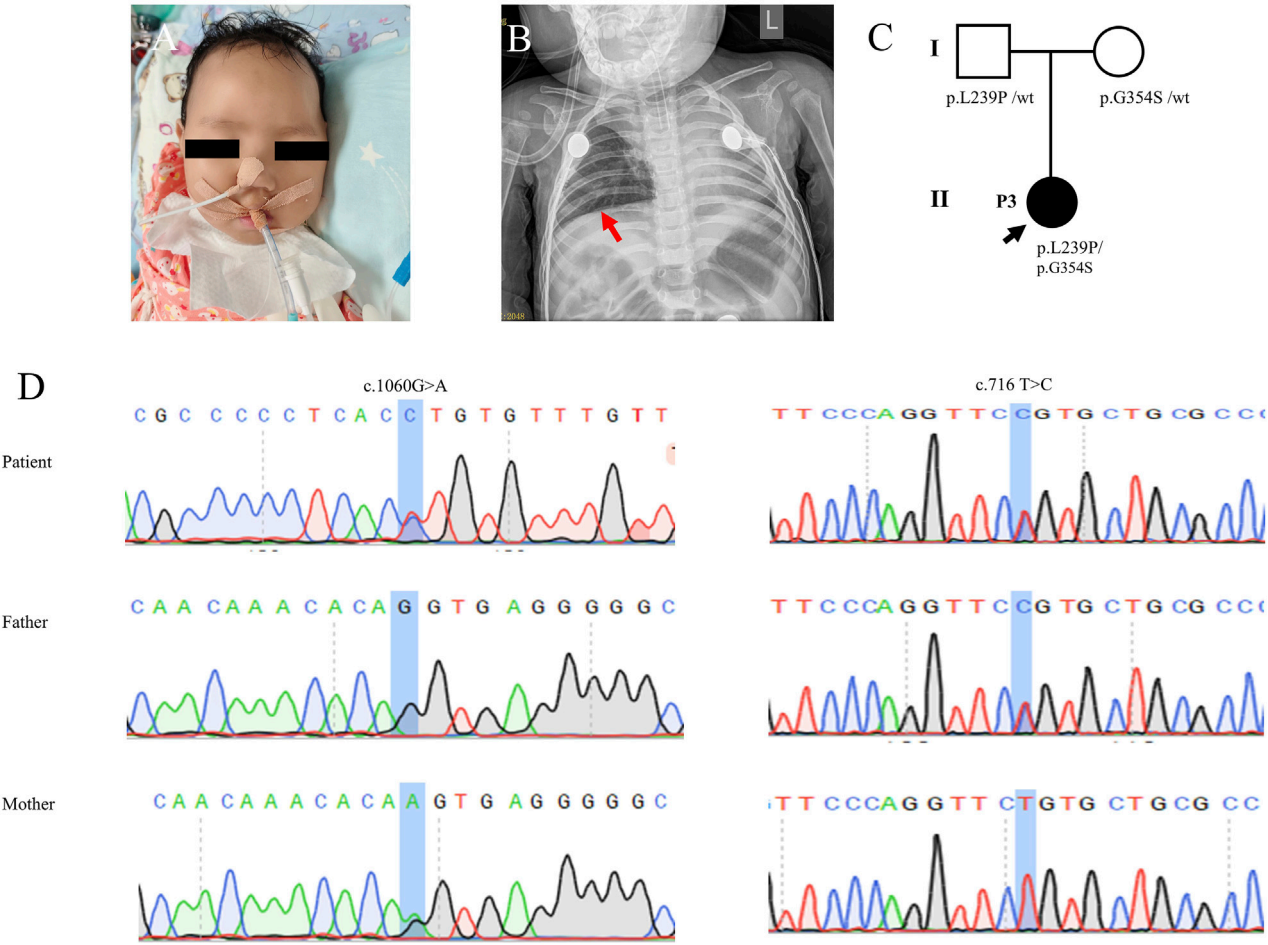
Clinical features and genetic results of P3. **(A)** Features of clinical symptoms. **(B)** Chest X-ray showed pneumonia and consolidation in the left lung but did not show diaphragm eventration. **(C)** Family pedigree. **(D)** Sanger sequecing results.

Physical examinations on admission showed that the infant exhibited a poor general condition, lethargic mental state, sigh breathing (down to 15 breaths/minute), tachycardia (ranging from 150 to 155 beats/minute), and SpO_2_ 78% under nasal cannula oxygen. She presented with gray face and cyanosis of lips, labored breathing, and suprasternal, intercostal, and subcostal retractions. Auscultation of the lungs showed coarse breath sounds in both lungs, and medium and fine wet rales and wheezing sounds could be heard. The infant presented with hypotonia, absent knee reflex, and negative pathological signs. The muscle strength grade was grade 2–3 for upper extremities and grade 0 for lower extremities (Figures 3A,B; Table 1).

Laboratory examinations showed that serum lactate, blood glucose, serum electrolytes, liver function, renal function, myocardial enzyme, and coagulation function were all in the normal range. Chest radiography revealed pneumonia and pulmonary mediastinal hernia, but no diaphragm eventration was observed. There were no abnormal findings in the electrocardiogram, electroencephalogram, echocardiography, or abdominal ultrasonography. Parainfluenza virus and *Klebsiella* sp. were detected from the respiratory tract.

The single-WES showed that the infant inherited compound heterozygous mutations in the *IGHMBP2* gene. Subsequently, all candidate mutations were confirmed by Sanger sequencing for the patients and her parents. The mutation c.1060G>A (p.G354S) was inherited from her mother, and the novel one c.716T>C (p.L239P) was inherited from her father (Figure 3C; Table 1). Her parents without clinical symptoms were confirmed as carriers of the mutations.

After 26 days of ventilator-assisted ventilation and anti-infection treatment, the patient’s lung function improved, but it was difficult to wean her from the ventilator. The child died the next day after her family made the decision to cease treatment.

### 3.2 Molecular detection by WES and bioinformatic analyses

**FIGURE 4:**
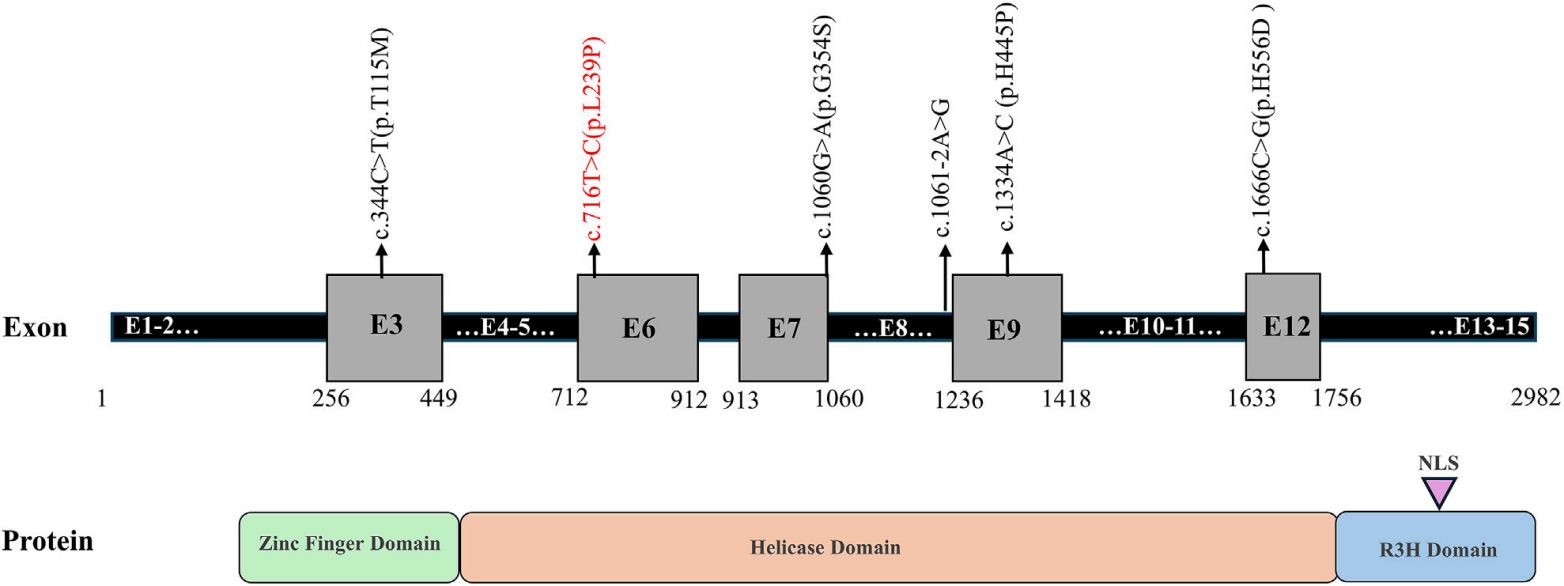
Schematic representation of the identified variants in the *IGHMBP2* gene. Note: novel mutation is shown in red.

A total of six mutations in the *IGHMBP2* gene, including five missense mutations and one splicing variant, were identified in this study (Figure 4; Table 2). Among them, four variants were classified as “Pathogenic or Likely pathogenic,” two as “Uncertain,” and one as “Likely benign,” according to the ACMG guidelines.

**TABLE 2:** Genetic data of the patients with SMARD1 and the prediction of the pathogenic effects of the variants in *IGHMBP2*.

Patient	Nucleotide change	Animo Acid change	Missense bioinformatics prediction			Splicing variants Prediction	ACMG
			SIFT	Polyphen 2	Mutation taster		
1	c.344C>T	p.T115M	Damaging	Possibly damaging	Polymorphism	No effect	Likely benign
	c.1060G>A	p.G354S	Damaging	Probably damaging	Disease-causing	No effect	Likely pathogenic
	c.1061–2A>G	—	—	—	Disease-causing	Affect	Pathogenic
2	c.1334A>C	p.H445P	Damaging	Probably damaging	Disease-causing	No effect	Pathogenic
	c.1666C>G	p.H556D	Tolerated	Benign	Disease-causing	No effect	Uncertain
3	c.716 T>C	p.L239P	Damaging	Probably damaging	Disease-causing	No effect	Uncertain
	c.1060G>A	p.G354S	Damaging	Probably damaging	Disease-causing	No effect	Likely pathogenic

Note:—, no data. Novel mutation is in bold.

**FIGURE 5:**
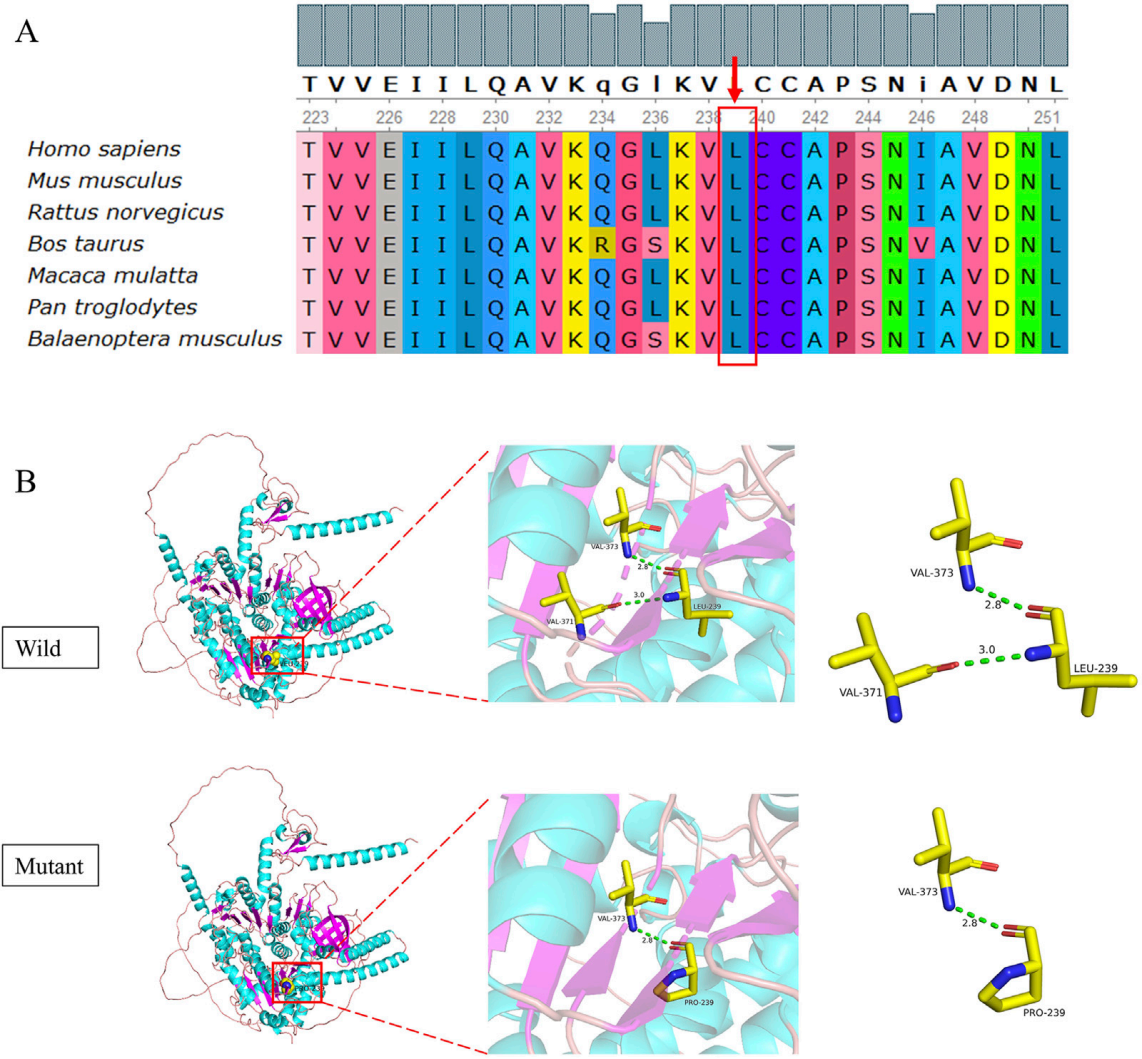
Conservation analysis **(A)** and 3D structure **(B)** of the novel mutation c.716T>C (p.L239P). The pink coil represents the loop structure, blue depicts an alpha-helix, and the purple region is a β-strand. Each color represents a different atom: yellow, C atom; gray, H atom; blue, N atom; red, O atom; and orange, S atom. The red dotted line represents the hydrogen bond.

A novel mutation c.716T>C (p.L239P) detected in patient 3 is a missense mutation that changes amino acid 239 from leucine to proline. In accordance with the ACMG guidelines, the novel mutation was preliminarily judged to be an uncertain variant (PM2_Supporting, PP3_Moderate). No record of the c.716T>C (p.L239P) mutation was found in the latest HGMD, and we were unable to acquire information about its pathogenicity. The result of bioinformatic analysis by SIFT, Polyphen2, and MutationTaster was indicative of a harmful consequence. The analysis of conservation showed that the amino acids at this site were highly conserved across multiple species, from fish to mammals (Figure 5A). As a change in the primary protein structure was caused by this novel missense variant, SWISS-MODEL was used to predict a potential alteration for the higher-order structure. In the protein structure model, a substitution of p.L239P in the helicase domain of the IGHMBP2 protein led to changes in hydrogen bonds. In the wild-type of the protein encoded by *IGHMBP2*, leucine at position 239 had two hydrogen bonds connected to valine at position 371 and 373. When leucine at position 239 was substituted by proline, the number of hydrogen bonds was reduced to 1, and it only linked to valine at position 373 (Figure 5B). These results all suggested that c.716T>C (p.L239P) had a high impact on protein function, leading to the SMARD1 phenotype. Because SMARD1 is an autosomal recessive disease, we proposed to change the original classification of “Unknown Significance” variant to “Likely Pathogenic” variant.

### 3.3 The disease etiology of the variants identified in this study

**FIGURE 6:**
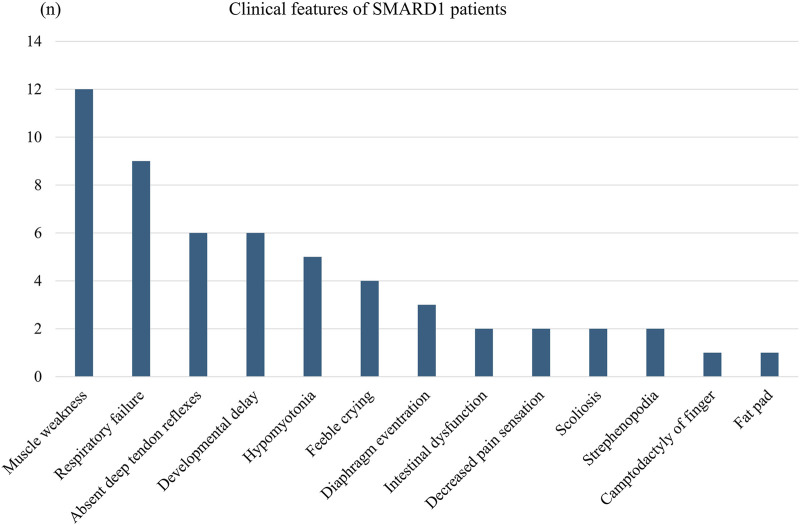
Clinical features of the SMARD1 patients.

We collated a detailed collection of clinical data from 12 patients with *IGHMBP2* variants identified in this study, including three patients from this study and nine patients from relevant literature (Table 3; Figure 6). There were two male and nine female patients. The proportion of compound heterozygotes and homozygotes were 83.3% (10/12) and 16.7% (2/12), respectively.

**TABLE 3:** Clinical and genetic features of 12 patients with *IGHMBP2* variants identified in this study.

Variants	PMID	Patient ID	Genetic defect		Age of onset	Age of diagnosis			Premature birth	Consanguinity/family	Clinical features	Tracheotomy	Status
			Genotype	Additional variants									
							Sex	Nationality		History			
c.344C>T	This study	Case 1	Comp	c.1060G>A/ c.1061–2A>G	7 m	1 y5 m	F	China	No	No/No	Diaphragm eventration, diaphragmatic paralysis, tachypnea, respiratory failure, absent knee jerk, hypomyotonia, delay in development, strephenopodia, intestinal dysfunction, and muscle weakness	No	Death at 1 y 6 m
	26922252	Case 2	Comp	c.1737C>A	4 m	4.5 y	F	China	No	No/No	Weakness in gastrocnemius and tibialis anterior, hypomyotonia, atrophy of the distal lower limb muscles, scoliosis, and absent Achilles tendon reflexes	No	Alive at least 4.5 y
	This study	Case 1	Comp	c.344C>T/c.10 61-2A>G							See above for details		
	This study	Case 3	Comp	c.716 T>C	Birth	9 m	F	China	No	No/No	Feeble crying, tachypnea, respiratory failure, absent knee jerk, hypomyotonia, delay in development, and muscle weakness	No	Death at 10 m
c.1060G>A	28397221	Case 4	Comp	c.344C>T/ c.2356delG	Birth	2 y1 m	F	China	No	No/No	Floppy infant, feeble crying, respiratory failure strephenopodia, hypomyotonia, and diaphragm eventration	No	Death at 3 y 1 m
	32154989	Case 5	Comp	c.2356delG	15 m	7 y	F	NA	NA	NA	History of easy falling and unsteady gait since walking began. Exam showed mild proximal girdle weakness but significant distal weakness with muscle wasting and mild tightness in Achilles tendons.	No	NA
c.1061–2A>G	This study	Case 1	Comp	c.344C>T/c.10 60G>A							See above for details		
	34539730	Case 6	Hom		3 m	3 m	M	China	Yes	NA	Feeble crying, intrauterine growth retardation, small for gestational age, limb muscle weakness, failure to thrive, tachypnea, respiratory failure, hyporeflexia, global developmental delay, and camptodactyly of finger	NA	NA
	This study	Case 7	Comp	c.1666C>G	50 d	5 m	F	China	No	No/No	Feeble crying, diaphragm eventration, tachypnea, respiratory failure, absent knee jerk, hypomyotonia, delay in development, intestinal dysfunction, and muscle weakness	No	Death at 7
		Case 8	Hom		4 m	NA	F		NA	NA	Intrauterine growth delay, cyanosis and dyspnea when crying, failure to thrive, respiratory distress, muscle weakness, absent deep tendon reflexes, and decreased pain sensation	15 m	Alive at least 4 y
c.1334A>C	38415210	Case 9	Comp	c.1235 + 3A > G	5 m	NA	F	Vietnam	NA	NA	Cannot move without support, dyspnea, failure to thrive, respiratory distress, and muscle weakness	No	Death at 8 m
		Case 10	Comp	c.1235 + 3A > G	4 m	NA	M		NA	NA	Leg muscle weakness, absent deep tendon reflexes, decreased pain sensation, and suspected scoliosis	No	Alive at least 3 y
		Case 11	Comp	c.1235 + 3A > G	2.5 m	NA	F		NA	NA	Respiratory failure and arm/leg/respiratory muscle weakness	No	Death at 2 y
	39170411	Case 12	Comp	c.1666C>G	2 m6 d	7 m	F	China	No	No/No	Frequent respiratory infections, respiratory failure, distal limb muscle weakness, and fat pad	No	NA
c.1666C>G	This study	Case 7	Comp	c.1334A>C							See above for details		
	39170411	Case 12	Comp	c.1334A>C							See above for details		
c.716 T>C	This study	Case 3	Comp	c.1060G>A							See above for details		

Note: d, day; m, month; y, year; F, female; M, male; NA, no data.

## 4 Discussion

Here, we described a case series of three patients with SMARD1. A previous study (Lesniak et al., 2024) revealed that the onset of respiratory distress is sometimes triggered by a respiratory tract infection, which is proven by our three cases. Among them, multiple pathogens from respiratory tract were identified in P2 and P3.

One of the diseases that should be differentiated from SMARD1 is 5q-SMA1, which is caused by homozygous deletion of exon 7 or exon 7 and 8 or a minor variation in the *SMN1* gene. The differentiating characteristics between them are shown in Table 4 (Keinath et al., 2021; Aponte Ribero et al., 2023), which aids to streamline the diagnostic process. The condition also needs to be distinguished from other early-onset neuromuscular disorders that cause motor retardation and softness in children, such as congenital myopathy, congenital and various muscular dystrophies, congenital myasthenic syndromes, and peripheral neuropathy. Most clinicians have little contact with SMARD1 and thus lack understanding of it. The clinical manifestations of the disease are diverse, and the early symptoms are not typical, which makes misdiagnosis easy. SMARD1 and other inherited neuromuscular diseases in infancy, especially 5q-SMA1, are characterized by progressive muscle weakness, hypotonia, absence of tendon reflexes, respiratory muscle weakness, and respiratory failure. It is difficult to distinguish it from other hereditary neuromuscular diseases in infancy. Genetic testing is an important method for clinical diagnosis of inherited rare diseases. Pedigree WES has the advantages of high-throughput, rapid, economical, and low missed diagnosis rate; it is also helpful for genetic counseling, which can achieve accurate diagnosis and treatment as soon as possible. With the rapid development of molecular diagnostic technology, an increasing number of neurogenetic diseases have been recognized. Genetic testing for such diseases should be performed as early as possible to avoid or reduce misdiagnosis and mistreatment.

**TABLE 4:** Comparison of the main features of SMARD1 and 5q-SMA type 1.

Features	SMARD1	5q-SMA type 1
Gene	IGHMBP2	SMN1
Inheritance pattern	Autosomal recessive	Autosomal recessive
	<3 m (early onset)	
Onset of symptoms	3–12 m (classical)	0–6 m
	>12 m (late onset)	
Decreased fetal movement	+	+
Fetal growth restriction	+	-
Muscle weakness distribution	Distal > proximal, lower limbs > upper limbs	Proximal > distal, lower limbs > upper limbs, the lower limbs were in a “frog leg” posture
Joint contractures	+ (primarily distal)	+
Scoliosis	+	-
Deep tendon reflexes	-	-
Respiratory insufficiency	+	+
The time of respiratory failure	Early stage of disease	Advanced stage of disease
Involvement of the diaphragm and intercostal muscles	Diaphragm > intercostal	Intercostal > diaphragm
Shape of thorax	Normal	Characteristic “bell-shaped” deformity
Foot deformity	+	-
Neurogenic changes in electromyography	+	+
Autonomic dysfunction		
Decreased pain sensitivity	+	-
Hyperhidrosis	+	-
Bladder and bowel dysfunction	+	-
Arrhythmia	+	-
Modifier gene	None	SMN2 copy number

Note: m, month; +, present; –, not present.

At present, the relationships between genotype and clinical type associated with the disorder are still not clearly elaborated. The extent of IGHMBP2 loss determines whether it develops into the mild type of CMT2S or the severe type of SMARD1. The onset ages of patient 1 of the first symptoms and respiratory failure were higher than in patient 2 and patient 3, which may suggest a mild clinical phenotype. Patient 1 harbored three variants in the *IGHMBP2* gene: two missense mutations including c.344C>T and c.1060G>A and one splicing mutation c.1061–2A>G. Among them, the variant c.344C>T is classified as “likely benign” according to ACMG. Three patients were previously reported harboring c.344C>T, including two cases with CMT2S who harbored one missense mutation besides c.344C>T (Shi et al., 2015; Yuan et al., 2017) and one with infantile SMARD1 presenting without respiratory involvement who harbored one missense mutation (c.1195G>A) and one splicing mutation (c.1060 + 5G>C) alongside c.344C>T (Luan et al., 2016).

So far, there are four patients including those in our study that harbored the missense variant c.1060G>A, and all of them were of Chinese origin, suggesting a hotspot in China. Among them, one case harbored compound heterozygous mutations c.1060G>A and c.2356delG (p.Arg786fs*45) and presented with CMT2S (Tsang et al., 2020), while three other cases, besides c.1060G>A, harbored c.344C>T/c.2356delG (Zhang et al., 2017), c.344C>T/c.1061–2A>G, and c.716T>C, respectively, and presented with SMARD1. Though the variant c.344C>T is SNP (rs32154989), when accompanied with one other mutation, it points to the mild disease type CMT2S, while with two other mutations, it points to the severe type SMARD1.

The clinical phenotypes caused by mutations in the *IGHMBP2* gene are significantly heterogeneous, and even the clinical phenotypes caused by the same variant are very different. For example, the same compound heterozygous mutation c.1235 + 3A > G/c.1334A > C manifests as CMT2S in some people and as SMARD1 in some others (Tran et al., 2024). Therefore, it is of great significance to report more cases of clinical phenotype–genotype correlation analysis for the exploration of gene pathogenicity, and it is also helpful for the early diagnosis of such patients, the selection of treatment options, and the evaluation of prognosis; it also provides an important basis for family genetic counseling.

A previous study (Lesniak et al., 2024) revealed that the overall survival of patients who developed symptoms after 3 months was significantly longer than that of children who developed symptoms within 3 months. In our study, all three cases refused tracheotomy and died soon after weaning from mechanical ventilation, so this conclusion cannot be tested.

SMARD1 patients have a poor prognosis, and there is no effective treatment at present. Currently, the clinical treatment is to prevent respiratory tract infection, provide mechanical ventilation to assist respiration, strengthen nutrition and other symptomatic treatment, prolong the survival time of children, and improve the quality of life. As more and more cases are reported, gene therapy will be gradually developed to solve the issues. IGHMBP2 protein is a DNA/RNA helicase of the SF1 superfamily involved in the regulation of pre-mRNA processing and transcription (Molnar et al., 1997). This protein includes a DNA helicase domain, a single-stranded nucleic acid-binding R3H motif, a zinc-finger region, a nuclear export signal region scattered in exons, and a nuclear localization signal region (Maystadt et al., 2004). The most reported variant located in the helicase domain has an ATP-dependent RNA/DNA helicase activity and is involved in the metabolism of RNA and DNA. In our study, 5/6 (83.3%) variants are located in the helicase domain, consistent with findings of a previous study. Since lower protein levels usually cause a more severe phenotype, increasing the expression level of functional IGHMBP2 protein may be a potential therapeutic target.

## 5 Conclusion

Our research highlights the clinical profiles across SMARD1 patients with the same genotype. Furthermore, we highlight the need for early genetic diagnosis to differentiate between muscle wasting disorders in patients with respiratory failure, regardless of whether diaphragmatic involvement is present. The identified novel mutation expands the genetic spectrum of the condition. The need for gathering clinical data to obtain genetic findings to deepen our understanding of genetic disorders is highlighted by our cases of the clinical manifestation of genetic variants that were previously thought to be uncertain.

## Data Availability

The original contributions presented in the study are publicly available. This data can be found here: https://ngdc.cncb.ac.cn/gsa-human/browse/HRA011916.
